# Study on effects of the train-induced airflow on the temperature field of single-track high-speed railway tunnels in cold regions

**DOI:** 10.1038/s41598-025-16068-4

**Published:** 2025-08-21

**Authors:** Jun Zhou, Liyuan Wu, Yan Gao, Xu Chen, Rongrong Liu

**Affiliations:** Huaiyin Institute of Technology, Huaian, 223003 China

**Keywords:** Cold-region engineering, Single-track tunnel, Train-induced airflow, Temperature field, Analytical solution, Engineering, Civil engineering

## Abstract

High-speed trains bring cold air into tunnels, significantly affecting the temperature field in cold-region tunnels, leading to risks of lining freeze-thaw damage, track deformation, and safety hazards, which is detrimental to thermal insulation design. Using the Kunlunshan Tunnel as a case study and based on unsteady flow theory, this study constructed the Navier-Stokes equations and proposed a theoretical calculation method for train-induced airflow. Dynamic mesh technology was adopted to develop an “equivalent wind speed” train-induced-airflow-temperature coupling computational model. The main idea of this model is to divide the effect of external wind speed on the tunnel into three distinct phases: the train-induced airflow phase, the residual airflow phase, and the natural wind phase. During each phase, the changes in wind speed are governed by the principle of conservation of cold air volume and converted into a steady airflow equivalent for that specific phase. This model was used to investigate the effects of train frequency, ground temperature of surrounding rock, external temperature, and external wind speed on the temperature field variation in single-track high-speed railway tunnels in cold regions. After verification, the temperature field inside the tunnel calculated by the “equivalent wind speed” method was in good agreement with the measured data, and can be used for subsequent numerical simulation calculations. The results show that when the interval between two trains is less than 20 min, the average temperature in the tunnel decreases by approximately 1.71 ℃ due to the significant impact of train-induced airflow. The impact of the ground temperature of surrounding rock on the longitudinal temperature gradient in cold-region tunnels ranges from 0.059 ℃ to 1.13 ℃ for every 100 m, and the ranges for external temperature and external wind speed are 0.080 ℃ to 2.286 ℃ and 0.002 ℃ to 1.134 ℃, respectively.

**Supplementary Information:**

The online version contains supplementary material available at 10.1038/s41598-025-16068-4.

## Introduction

As China’s railway industry develops rapidly, the construction of railway networks has expanded into colder regions. High-speed railway tunnels located in high-latitude and high-altitude areas face varying degrees of frost damage during operation. The heat transfer between the cold air outside the tunnel and the air, surrounding rock, and lining structures inside the tunnel results in a significant temperature drop in the tunnel. This is the primary cause of frost damage in tunnels located in cold regions. During the operation of high-speed railways, the train-induced airflow exacerbates frost damage by carrying cold air into the tunnel^1^.

Methods for calculating train-induced airflow have been extensively studied. For example, Wang et al.^2^ used one-dimensional theoretical analysis to deduce a simple calculation method for piston wind speed and airflow distribution. Zhang et al.^3^ developed a theoretical method based on the Bernoulli equation to calculate train-induced airflow in subway tunnels and validated its accuracy and feasibility. Liu et al.^4^ analyzed the energy conservation of subway tunnel ventilation systems in various cities in China using piston wind and identified the optimal train speed for reducing energy consumption. Lanchava. O et al.^5^ found that the piston effect in subway tunnels is influenced by factors such as train speed and the geometry of a tunnel and a train, and the velocity of airflow generated by the piston effect is proportional to the train speed and the degree of fill rate of a tunnel. Zeng et al.^6^ modified the theoretical calculation method for the piston wind action coefficient based on field test results and numerical simulations. Duan et al.^7^, based on the three-dimensional transient compressible Reynolds-averaged the Navier-Stokes equations and the k - ω Shear Stress Transport turbulence model and using overset grid technology studied the effect of line spacing on the aerodynamic drag, lateral force, and lift experienced by high-speed trains during their passage in tunnels at different speeds. Huang et al.^8^ investigated the transient pressure and aerodynamic forces induced by a train passing through the tunnel under the conditions of the inclined shaft open without and with air intake in the Wushaoling Tunnel. Current theoretical research on train-induced airflow mainly focuses on the changes in air pressure and the velocity of train-induced airflow inside the tunnel during the train’s passage, and the impact of train-induced airflow on the temperature field has been insufficiently studied.

Only a few studies have been conducted on the impact of train-induced airflow on the temperature field. For example, Zhao et al.^9^ investigated the differences in longitudinal temperature distribution of tunnels in cold regions during winter and summer, and proposed that the temperature changes of the air inside the tunnel caused by traffic wind are transient. Tao et al.^10^ developed a three-dimensional unsteady heat transfer model of the surrounding rock and investigated the relationship between the piston wind in cold-region tunnels and the anti-freezing length. Gao et al.^11^ established a model test system to investigate the influences of the temperature outside the tunnel and train operation conditions on the length of the tunnel’s negative-temperature length. Tao et al.^12^, through theoretical analysis and numerical simulations, studied the distribution of the temperature field inside long cold-region railway tunnels under the influence of train piston wind. The effects of natural wind and mechanical ventilation on the temperature field inside tunnels have also been explored. For example, Tan et al.^13^ conducted a study using the standard $k - \varepsilon$ model, wall function, thermal function, and adaptive finite element method to develop an efficient computational fluid dynamics (CFD) approach for simulating gas flow and air-rock heat transfer characteristics within cold-region tunnels, and compared the results with experimental data. Tan^14^ established a temperature field model and performed numerical simulations to investigate the impacts of airflow on temperatures in cold-region tunnels and surrounding rocks. Accordingly, they suggested the use of insulation materials for tunnel thermal protection. Zeng et al.^15^ performed a model test and finite difference calculations to analyze the effects of inlet temperatures and ventilation velocities on temperature fields in railway tunnels. They found that low-temperature airflow and positive ventilation direction are directly proportional to tunnel frost damage, while negative ventilation direction is inversely proportional to it. Tao et al.^16^ developed a numerical heat transfer model of a cold-region tunnel and found that mechanical ventilation could effectively reduce the range of ambient temperature below 0 ℃ in tunnels. They also investigated the effects of jet fan operation time and natural airflow velocity on the temperature field. Jame et al.^17^, based on the principles of fluid dynamics, heat transfer, and aerodynamics, studied the temperature field of the Galongla Tunnel in Tibet under ventilation effects and determined the required length for thermal protection. Guo et al.^18^ analyzed the impacts of natural wind, annual average temperature, and annual temperature fluctuations on the temperature field in the Qilianshan Tunnel through environmental monitoring inside and outside the tunnel. However, few theoretical studies have been conducted on the impact of train-induced airflow on the temperature field in cold-region high-speed railway tunnels.

This study, taking the Kunlunshan Tunnel as a case study, constructed the Navier-Stokes equations based on unsteady flow theory and proposed a theoretical calculation method for train-induced airflow. Dynamic mesh technology was adopted to develop an “equivalent wind speed” train-induced-airflow-temperature coupling computational model. The main idea of this model is to divide the effect of external wind speed on the tunnel into three distinct phases: the train-induced airflow phase, the residual airflow phase, and the natural wind phase. During each phase, the changes in wind speed are governed by the principle of conservation of cold air volume and converted into a steady airflow equivalent for that specific phase. This model was used to investigate the effects of train frequency, ground temperature of surrounding rock, external temperature, and external wind speed on the temperature field variation in single-track high-speed railway tunnels in cold regions.

## Calculation theory for Train-Induced airflow

Due to the complexity of investigating the impact of train-induced airflow on the temperature field in cold-region tunnels, the following assumptions were made to facilitate the derivation of a complete analytical solution:

A. The air inside and outside the tunnel is incompressible.
B. The air inside the tunnel is considered an ideal gas, and the gas flow is assumed to be one-dimensional.
C. The tunnel cross-section does not change, meaning changes in tunnel cross-section, such as those caused by passing bays, are ignored during derivation.
D. The initial wind speed inside the tunnel is 0 before the train enters, with the influence of natural wind neglected.
E. The train travels at a constant speed.

### Computational model for Train-Induced airflow

**Fig. 1:**
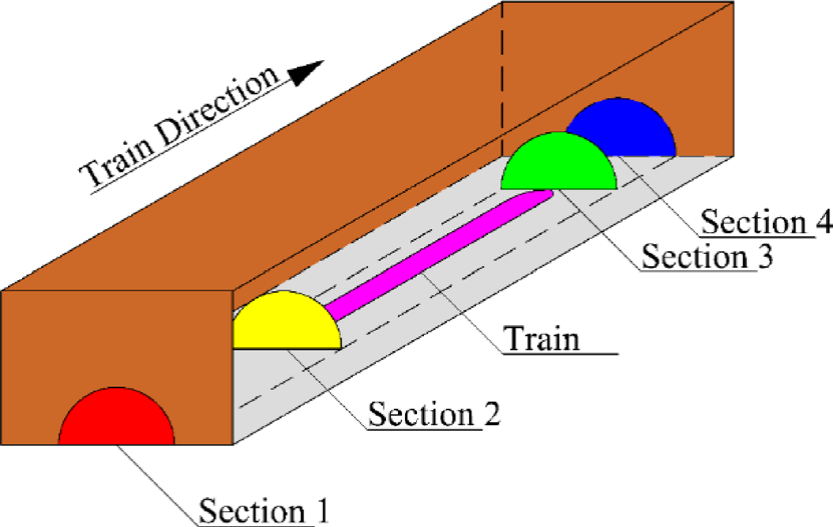
Train traveling after completely entering the tunnel.

According to Shen’s study on train-induced airflow^19^, after the train completely enters the tunnel, it travels at a constant speed ${v_{tr}}$. As shown in Fig. 1, Sect. 1 and Sect. 4 represent the tunnel’s entrance and exit, respectively. Section 2 and Sect. 3 are located at the front and rear of the train and move with the train as it travels.${L_{tr}}$ is the length of the train (m), $w$ is the airflow velocity in the annular region (m/s), ${v_0}$is the speed of the train-induced airflow inside the tunnel at the front and rear ends of the train (m/s), and ${L_{tu}}$ is the length of the tunnel (m).

The cross-sectional areas of the tunnel and train are${A_{tu}}$and${A_{tr}}$, respectively. The volume of air displaced by the train during a period ${\text{d}}t$ can be expressed as ${A_{tr}}{v_0}{\text{d}}t$, Of this, a portion flows toward the front of the train with a volume of ${A_{tu}}{v_1}{\text{d}}t$,while the remaining portion $({A_{tu}} - {A_{tr}})w{\text{d}}t$ flows into the annular region between the train and the tunnel walls. The continuity equation is therefore:

1 $${v_1}({A_{tu}} - {A_{tr}})+{v_{tr}}{A_{tr}}={v_0}{A_{tu}}$$

where ${v_0}$ refers to the speed of the train-induced airflow inside the tunnel at the front and rear ends of the train, while ${A_{tu}}$ and ${A_{tr}}$ are the cross-sectional area of the tunnel and the train, and ${v_{tr}}$ is the speed of the train;

Using the Bernoulli equation, the pressure relationship between adjacent sections is derived. The pressure relationship between Sects. 1 and 2 is:

2 $${P_1}={P_2}+\frac{{\rho v_{0}^{2}}}{2}+{K_1}\frac{{\rho v_{0}^{2}}}{2}+{K_{1 - 2}}\frac{{\rho v_{0}^{2}}}{2}$$

where ${P_1}$ and ${P_2}$ are the pressure of Sects. 1 and 2, while $\rho$is the density of air, ${K_1}$ and ${K_{1 - 2}}$ are the local resistance coefficient at the entrance of the tunnel and the local resistance coefficients between Sect. 1 and Sect. 2.

between Sects. 2 and 3 is:

3 $${P_3} - {P_2}={K_m}\frac{{\rho {{({v_{tr}} - {v_0})}^2}}}{2}$$

where ${P_2}$ and ${P_3}$ are the pressure of Sects. 2 and 3, ${K_m}$ is the piston action coefficient of the train.

between Sects. 3 and 4 is:

4 $${P_3}+\frac{{\rho v_{0}^{2}}}{2}={P_4}+{K_4}\frac{{\rho v_{0}^{2}}}{2}+{K_{3 - 4}}\frac{{\rho v_{0}^{2}}}{2}$$

where ${P_4}$is the pressure of Sect. 4, ${K_4}$ and ${K_{3 - 4}}$ are the local resistance coefficients at the exit of the tunnel and the local resistance coefficient between Sect. 3 and Sect. 4.

By combining these equations, the relationship between Sects. 1 and 4 is obtained, leading to the equation for train-induced airflow:

5 $${v_0}=\frac{{{v_{tr}}}}{{1+\sqrt {\frac{{{K_1}+{K_4}+\frac{{{\lambda _0}({L_{tu}} - {L_{tr}})}}{{{d_0}}}}}{{{K_m}}}} }}$$

## Computational model for residual airflow

To describe how the velocity of train-induced airflow gradually reduces to the natural wind speed after the train completely leaves the tunnel, basic principles from fluid mechanics, especially those related to momentum transfer, can be applied. In this study, we used the simplified form of the Navier-Stokes equations, which describe Newtonian viscous fluid flow.

**Fig. 2:**
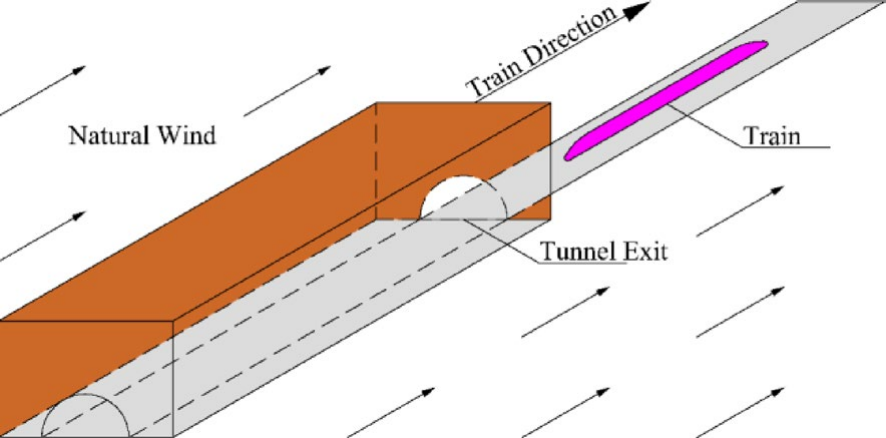
Train traveling after completely leaving the tunnel.

Figure 2 illustrates the situation after the train completely leaves the tunnel. First, assume that the air density is $\rho$, the dynamic viscosity coefficient of the air is $\mu$, and the train speed is ${v_{tr}}$. It is also assumed that, due to the train’s motion, a small vortex region is formed behind the train with an initial velocity of ${v_0}$. The goal is to determine the time required for the velocity of train-induced airflow to reduce from the initial value ${v_0}$ to the natural wind speed ${v_1}$.

Assume that the airflow inside the tunnel can be approximated as one-dimensional, incompressible, and viscous. In this case, a simplified form of the Bernoulli equation that considers the frictional terms caused by viscosity can be used to describe the airflow velocity reduction process.

A. Expression for the reduction process.

For one-dimensional flow, a simplified form of the momentum equation can be expressed as:

6 $$\rho A\frac{{dv}}{{dt}}= - \frac{1}{2}\rho f{v^2}A - \rho gh\frac{{\partial h}}{{\partial x}}$$

where $v$ is the airflow velocity, $f$ is the friction coefficient, $g$ is the gravitational acceleration, and $h$ is the height (which is considered constant inside the tunnel, so $\frac{{\partial h}}{{\partial x}}=0$)

Since the focus is on airflow velocity reduction, the gravitational term can be neglected. It is also assumed that the tunnel wall roughness is uniform, so the friction coefficient $f$ is constant. Therefore, the equation simplifies to:

7 $$\frac{{dv}}{{dt}}= - \frac{1}{2}f{v^2}$$

This is a nonlinear differential equation regarding $v$, with an initial value of ${v_0}$. The expression for airflow velocity reduction over time is:

8 $$v(t)=\frac{{{v_0}}}{{1+\frac{1}{2}f{v_0}t}}$$

B. Expression for the time required for reduction.

To obtain the time $\Delta t$ required for the wind speed to reduce from ${v_0}$​ to ${v_1}$​,$v(t)={v_1}$​ is substituted into the equation above:

9 $${v_1}=\frac{{{v_0}}}{{1+\frac{1}{2}f{v_0}t}}$$

By solving the equation, we obtain:

10 $$\Delta t=\frac{2}{{f{v_0}}}(\frac{{{v_0}}}{{{v_1}}} - 1)$$

where $\Delta t$ is the time required for the velocity of train-induced airflow to reduce to the natural wind speed.

The expression for the time needed for the reduction from ${v_0}$ to ${v_1}$ can be obtained.

## Numerical simulation

### Case study

**Fig. 3:**
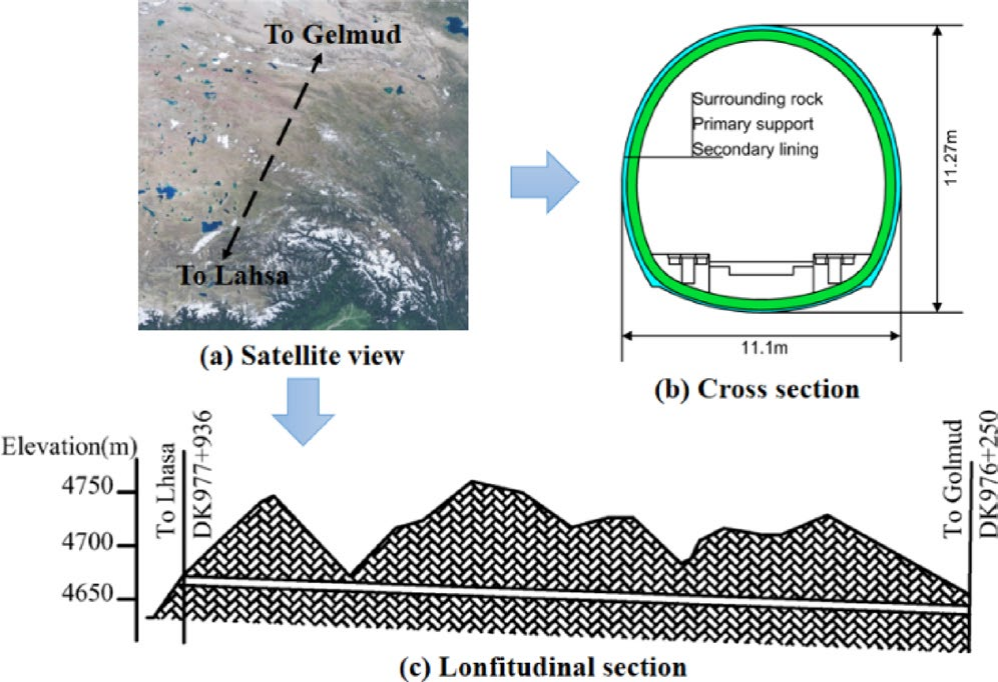
Schematic diagrams of the Kunlunshan Tunnel. (The satellite image was generated by the web version of Baidu Maps. The website link is https://map.baidu.com/dir/%E6%8B%89%E8%90%A8%E5%B8%82/%E6%A0%BC%E5%B0%94%E6%9 C%A8%E5%B8%82/@10353506.99960068,3874641.6387991863,7.85z/maptype%3DB_EARTH_MAP?querytype=bt&c=1&sn=1$$a887fc00209a4f4bb05c6ae6$$10150050.00,3438517.00$$%E6%8B%89%E8%90%A8%E5%B8%82$$0$$$$&en=1$$6eed59d082e57f532e4733ea$$10568043.00,4331913.00$$%E6%A0%BC%E5%B0%94%E6%9 C%A8%E5%B8%82$$0$$$$&sc=1&ec=1&pn=0&rn=5&exptype=dep&exptime=2025-07-30%2017:50&version=5&da_src=shareurl).

The Kunlunshan Tunnel is a single-track railway tunnel, starting at Lahsa and ending at Golmud, with a total length of 1686 m. This tunnel is located in the permafrost region at the northern edge of the Qinghai-Tibet Plateau, in a mid-to-high-altitude mountainous area. The slope on the entrance side faces the shaded side, with a maximum frozen soil depth of approximately 2.7 m, while the slope on the exit side faces the sun, with a maximum frozen soil depth ranging from 2.1 m to 3 m. The temperature in the area undergoes extreme fluctuations, with the highest occurring in July and the lowest in January. The annual average ambient temperature is -4.27 ℃, and the record high and low temperatures stand at 23.7 ℃ and − 37.7 ℃, respectively. The prevailing wind direction is from the west, with the maximum wind speed reaching 23.0 m/s. Figure 3 (a) shows a satellite view of the entire Kunlunshan Tunnel, Fig. 3 (b) presents the cross-sectional view of the tunnel, and Fig. 3 (c) illustrates the longitudinal section of the tunnel.

## Numerical calculation model

### Geometry model

Based on the Kunlunshan Tunnel, a three-dimensional computational model of the temperature field in the cold-region tunnel under the influence of train-induced airflow was established. The 8-car CRH380 high-speed train, the research object, has a width of W = 3.3 m, a height of H = 3.7 m, and a length of ${L_{tr}}$= 200 m. The cross-sectional area of the train is 9.11${m^2}$. The train is simplified to a smooth car body, without simulating details such as the bogie and windshields. According to the Code for Design of High-Speed Railway (TB 10621 − 2014), the clearance area for a single-track tunnel is 70${m^2}$. The tunnel length is ${L_{tu}}$= 1686 m, and the blockage ratio when the train passes through the tunnel is $\beta$= 0.112.

### Boundary conditions and mesh division

A full-scale high-speed train model was established in the numerical simulation. The simulation used an 8-carriage CRH380 EMU train, with dimensions of 3.7 m (height) × 3.3 m (width) × 200 m (length). The train was modeled exactly according to the factory drawings at a 1:1 scale. The computational coordinate system was centered on the train’s center, with the tunnel longitudinal direction as the X-axis, the transverse direction as the Y-axis, and the vertical direction as the Z-axis.

A computational domain that includes both the air inside the tunnel and the air outside the tunnel’s entrance and exit was created. The train’s height of 3.7 m was taken as the characteristic height H; the ratio of the cross-sectional area of the train to the computational domain was less than 0.01. The height and width of the computational domain of air were both set to 10 H, with upstream length L1 equal to 16 times the characteristic height and downstream length L2 equal to 80 H, which is no less than one train module length. This ensures that the external computational domain does not influence the flow at the tunnel entrance. The tunnel is a standard single-track tunnel with a lining thickness of 0.5 m, surrounding rock thickness of 10 m, radius of 6.65 m, and height of 8.5 m. The computational model adopts hexahedral mesh discretization. The external computational domain contains 8 million mesh cells, and the train region contains 20 million mesh cells, with a minimum mesh quality of 0.7 and an overall mesh quality no less than 0.9.

**Fig. 4:**
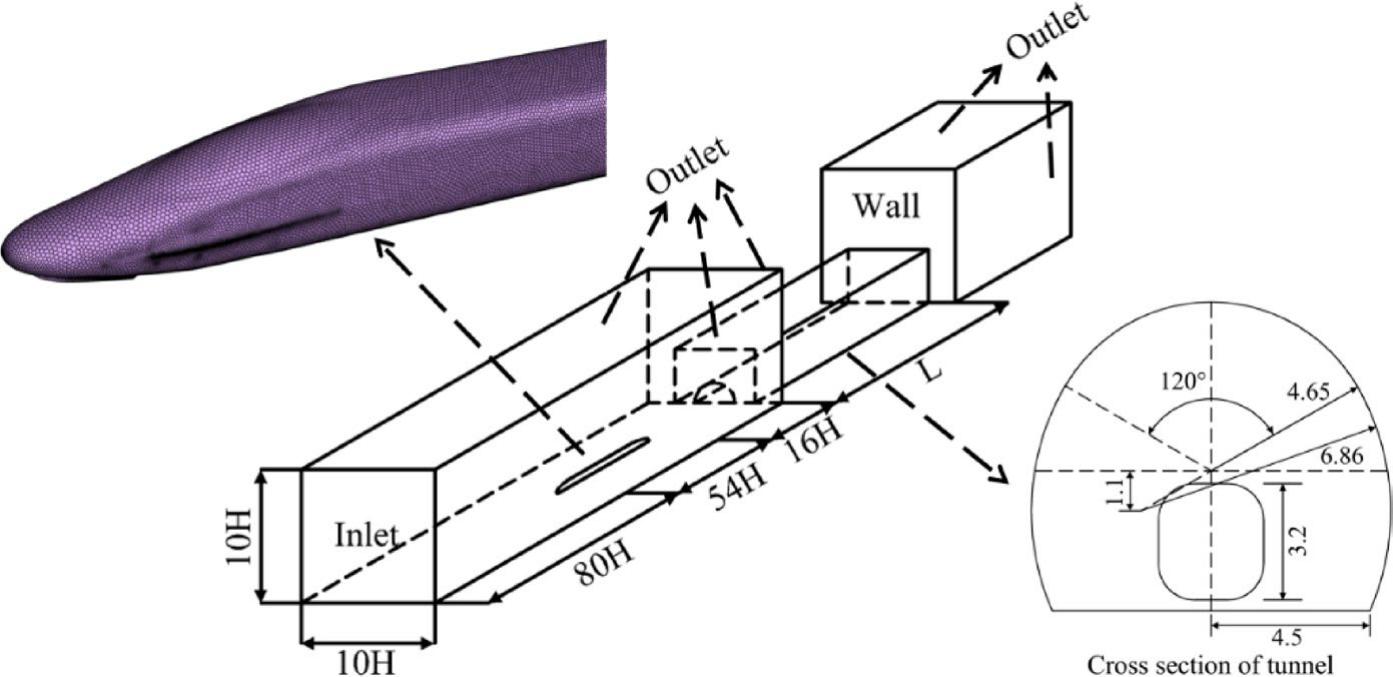
Boundary conditions of the model.

The Dynamic Mesh method was employed to simulate the process of a train passing through the tunnel. Interfaces between the tunnel, the surrounding rock, and the lining were used for information exchange. A uniform velocity inlet boundary condition (Velocity-Inlet) was applied at the tunnel entrance, a Pressure-Outlet boundary condition was applied at the tunnel exit, and a Pressure Far-field boundary condition was used for the air domain at the train entrance. The train, the surrounding rock, and the tunnel walls were all set as Wall boundary conditions. Figure 4 presents the schematic diagram of the computational model.

### Parameter settings

A. Air, Lining, and Surrounding Rock Parameters

The monthly average air temperature outside the Kunlunshan Tunnel can be approximated by the following sinusoidal curve^20^:

11 $$T=12\sin (\frac{{2\pi }}{{8640}}t+\frac{3}{2}\pi ) - 3.6$$

where $T$ is the monthly average air temperature outside the Kunlunshan Tunnel, in Celsius (${}^{ \circ }C$), and $t$ is time, in months.

The frozen soil characteristics and thermal parameters for the tunnel site are presented in Table 1.

**Table 1:** Model calculation Parameters.

Material	Density ($kg \cdot {m^{ - 3}}$)	Specific Heat Capacity ($J \cdot {(kg \cdot {}^{ \circ }C)^{ - 1}}$)	Thermal conductivity ($W \cdot {(m \cdot k)^{ - 1}}$)
Air	1.23	1.003	0.02
Lining	2500	0.92	1.74
Surrounding rock	1700	1.021	2.3

During the calculation process, the air was considered an ideal gas, and the air density was set according to the ideal gas law. The air dynamic viscosity coefficient was taken as $1.7894 \times {10^{ - 5}}kg/m \cdot s$.

B. Convective Heat Transfer Coefficient

The temperature field in the tunnel under the influence of negative airflow involves a coupled field of both fluid and solid media. This coupled field consists of two parts: the temperature field of the fluid and the temperature field of the solid. These two parts transfer heat through their contact surface. Therefore, in the numerical calculation, the heat conduction between the air and the tunnel’s internal structure and surrounding rock is primarily considered.

When the train is moving, the convective heat transfer coefficient between the air and the tunnel walls has a significant impact, so the variation of the coefficient must be considered. It can be calculated via the following equation^21^:

12 $$h=0.023\frac{\lambda }{d}{\left( {\frac{{ud}}{v}} \right)^{0.8}}P{r^{0.3}}$$

Where $\lambda$ is the thermal conductivity coefficient of the train-induced airflow in the tunnel(W·(m·K)^−1^; $d$ is the hydraulic diameter of the tunnel (m);$u$ is the velocity of train-induced airflow at the tunnel wall (m/s); $v$ is the kinematic viscosity (${{\text{m}}^{\text{2}}} \cdot {{\text{s}}^{ - {\text{1}}}}$), taken as 1.637e^− 5^; and $Pr$ is the Prandtl number, taken as 0.7. Table 2 provides the calculation for the convective heat transfer coefficient.

**Table 2:** Convective heat transfer coefficient.

$u/(m/s)$	$\lambda {\text{/(W}} \cdot {({\text{m}} \cdot {\text{K)}}^{{\text{-1}}}})$	${\text{h/(W}} \cdot {{\text{(}}{{\text{m}}^{\text{2}}} \cdot {\text{K)}}^{ - {\text{1}}}})$
15	2.3e^− 2^	23.38
5	2.3e^− 2^	9.71
1.5	2.3e^− 2^	3.71

### Calculation method for equivalent wind speed

**Fig. 5:**
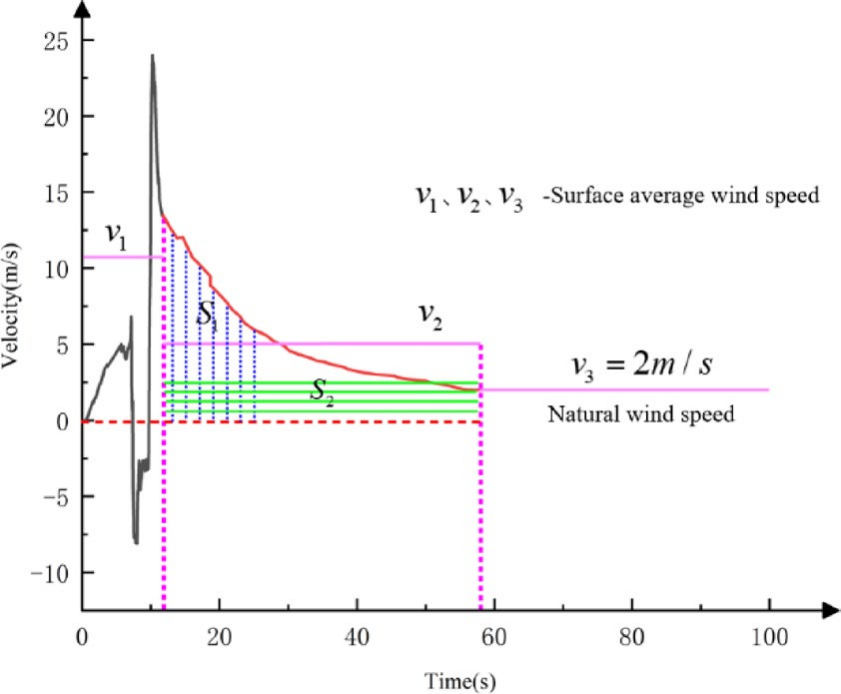
The “Equivalent Wind Speed” Theoretical Calculation Method.

Due to the large number of mesh cells in the computational model, reaching 28 million, the calculation process is time-consuming (in years) and computationally expensive. To facilitate the calculation of the temperature field in cold-region tunnels under the influence of train-induced airflow, an equivalent calculation method was proposed. The main idea is to divide the effect of external wind speed on the tunnel into three phases: the train-induced airflow phase, the residual airflow phase, and the natural wind phase. During these phases, the changes in wind speed are governed by the principle of conservation of cold air volume and converted into a steady airflow that enters the tunnel during the corresponding phase. The equivalent wind speed calculation is shown in Fig. 5.

The three-phase equivalent wind speed theoretical calculation method is as follows.

A. Train Passage Phase.

The velocity of train-induced wind ${v_0}$ when the train is fully inside the tunnel can be derived through Eq. (5):

$${v_0}=\frac{{{v_{tr}}}}{{1+\sqrt {\frac{{{K_1}+{K_4}+\frac{{{\lambda _0}({L_{tu}} - {L_{tr}})}}{{{d_0}}}}}{{{K_m}}}} }}$$

Assuming the train completely enters the tunnel at time ${t_1}$, the duration $\Delta {t_1}$ for the train to be fully inside the tunnel is expressed as:

13 $$\Delta {t_1}=\frac{{{L_{tu}} - {L_{tr}}}}{{{v_{tr}}}}$$

The cold air volume ${V_1}({m^3})$ entering the tunnel during this phase is:

14 $$\begin{array} {l} V_{1} = A_{{tu}} \int_{{t_{1} }}^{{t_{1} + \Delta t_{1} }} {v_{0} dt} \\ \quad\,\,\, = A_{{tu}} \int_{{t_{1} }}^{{t_{1} + \Delta t_{1} }} {\frac{{v_{{tr}} }}{{1 + \sqrt {\frac{{K_{1} + K_{4} + \frac{{\lambda _{0} (L_{{tu}} - L_{{tr}} )}}{{d_{0} }}}}{{K_{m} }}} }}dt} \\ \quad\,\,\, = \frac{{A_{{tu}} v_{{tr}} (L_{{tu}} - L_{{tr}} )}}{{v_{{tr}} [1 + \sqrt {\frac{{K_{1} + K_{4} + \frac{{\lambda _{0} (L_{{tu}} - L_{{tr}} )}}{{d_{0} }}}}{{K_{m} }}]} }}\end{array}$$

B. Residual Airflow Phase.

From Eqs. (8) and (10), the equation for cold air volume reduction and the time required for the velocity reduction to the natural wind speed can be obtained. Assuming the train completely exits the tunnel at time ${t_2}$, the duration of the residual wind is $\Delta {t_2}$. The cold air volume ${V_2}({m^3})$ entering the tunnel during this phase is:

15 $$\begin{array} {l} V_{2} = A_{{tu}} \int_{{t_{2} }}^{{t_{2} + \Delta t_{2} }} {v(t)dt} \\ \quad\,\,\, = A_{{tu}} \int_{{t_{2} }}^{{t_{2} + \Delta t_{2} }} {\frac{{v_{0} }}{{1 + \frac{1}{2}fv_{0} t}}dt} \\ \quad\,\,\, = A_{{tu}} \cdot \Delta t_{2} \cdot \frac{2}{f}\ln (1 + \frac{1}{2}fv_{0} t_{2} ) \end{array}$$

The equivalent wind speed ${v_{e2}}$ during this phase is calculated as:

16 $${v_{e2}}=\frac{V}{{{A_{tu}} \cdot \Delta {t_2}}}=\frac{2}{f}\ln (1+\frac{1}{2}f{v_0}{t_2})$$

C. Natural Wind Phase.

When there is no train in the tunnel, and the residual airflow induced by the previous train has reduced to the natural wind speed, it is assumed that external natural wind enters the tunnel at a constant speed. The natural wind speed is taken as 2 m/s. The equivalent wind speed calculation for this phase is:

17 $${v_{e3}}=2m/s$$

### Simulation results validation

**Fig. 6:**
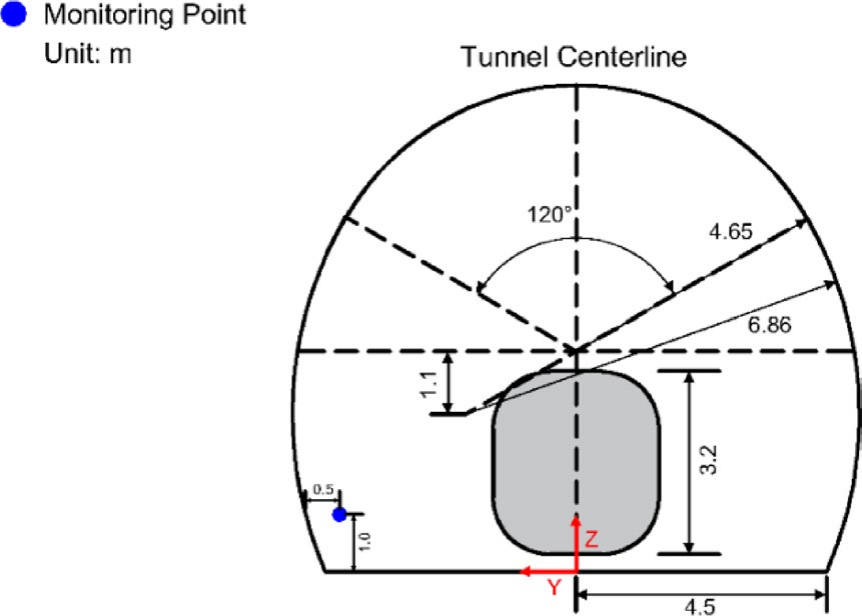
Monitoring point arrangement.

To validate the effectiveness of the numerical simulation of train-induced airflow during train passage through a tunnel, the simulation results were compared with experimental data from Pope et al.^22,23^, who performed experiments on train-induced airflow in a tunnel in the UK. A tunnel and train model identical to that used in the tests done by Pope et al. was established, with the same monitoring point arranged. The monitoring point was located 150 m from the tunnel entrance, 1 m above the ground, and 0.5 m from the sidewall, as shown in Fig. 6.

**Fig. 7:**
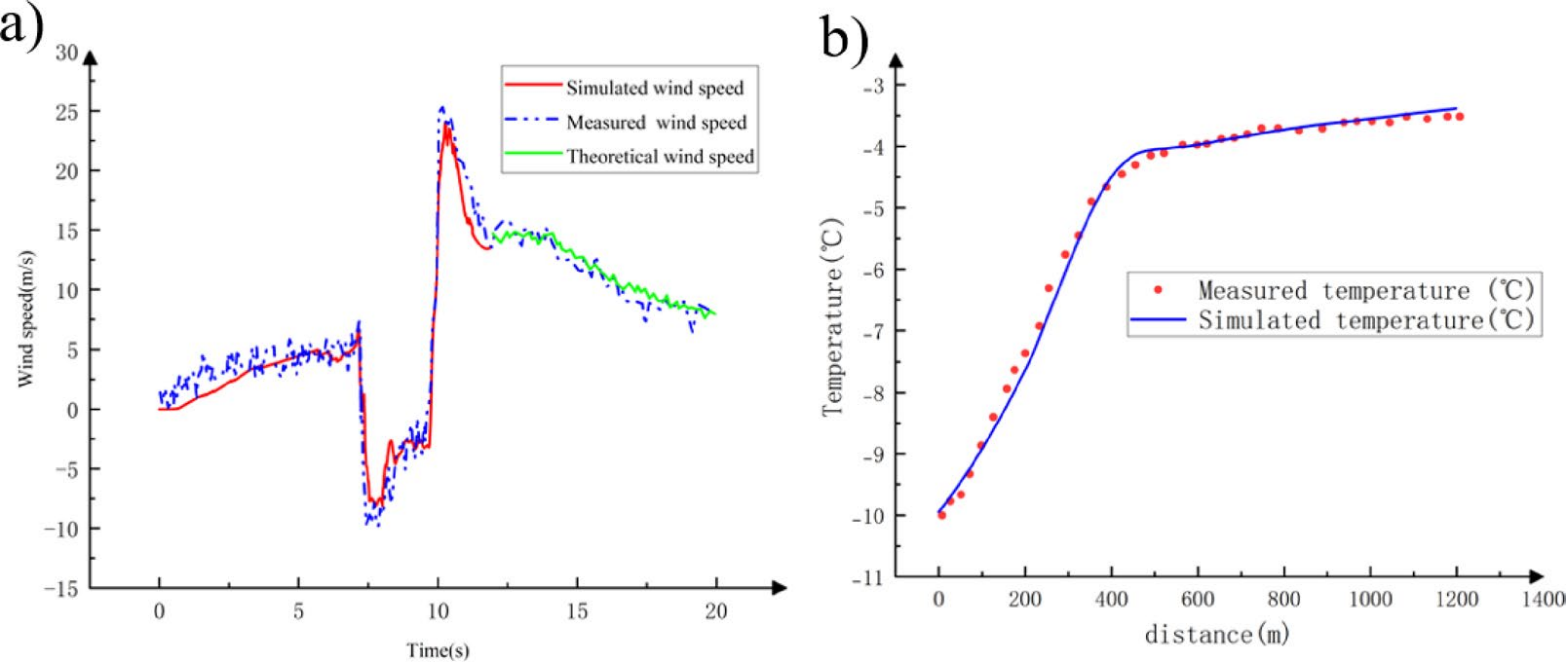
(**a**) Comparison of the theoretical, simulated, and measured train-induced airflow speed variations and (**b**) Comparison of simulated and measured temperature field with the equivalent wind speed.

Figure 7 (a) presents a comparison of the theoretical, simulated, and measured train-induced airflow speed variations. In the numerical simulation, the maximum positive peak and the maximum negative peak of the airflow speed were 23.804 m/s and − 8.254 m/s, respectively, with relative errors of 0.603% and 1.05% compared to the experimental data. The theoretical derivation results of the velocity of residual train-induced airflow and the simulated results were in good agreement. Therefore, the theoretical derivation and numerical simulation method for train-induced airflow were applicable for subsequent analysis.

Figure 7 (b) illustrates the variation law of the longitudinal temperature field in the tunnel obtained through numerical simulation using the equivalent wind speed method. To verify the effectiveness of the numerical simulation of train-induced airflow during train passage, the computational results were compared with experimental data from Lu et al.^24^ at the Anshan Tunnel of the Harbin-Dalian High-Speed Railway in China.

In summary, as the numerical simulation results of the tunnel temperature field using the equivalent wind speed method agreed well with the experimental data, the “equivalent wind speed” method could be used for related calculations and analyses of the coupling effect between train-induced airflow and the temperature field.

## Results and discussion

### Impact of different train frequencies on the temperature field

Based on the operation of the Harbin-Dalian Railway in China, during off-peak times, the interval between two trains typically ranges from 30 min to 1 h during the day. During late-night periods, train frequencies are reduced, with intervals extending to 1–2 h. During peak periods, such as the Spring Festival or summer holidays, train frequencies increase significantly, and the interval can shorten to 10–30 min.

Using the method of controlling variables, with field data from the Kunlunshan Tunnel as boundary conditions for the calculation, the ground temperature of the surrounding rock was set to 6 ℃, external temperature to -15 ℃, and external wind speed to 2 m/s, with a train speed of 300 km/h. The temperature field variations in the tunnel under natural ventilation conditions, and with trains passing through the tunnel at intervals of 5, 10, 15, and 20 min, were simulated.

**Fig. 8:**
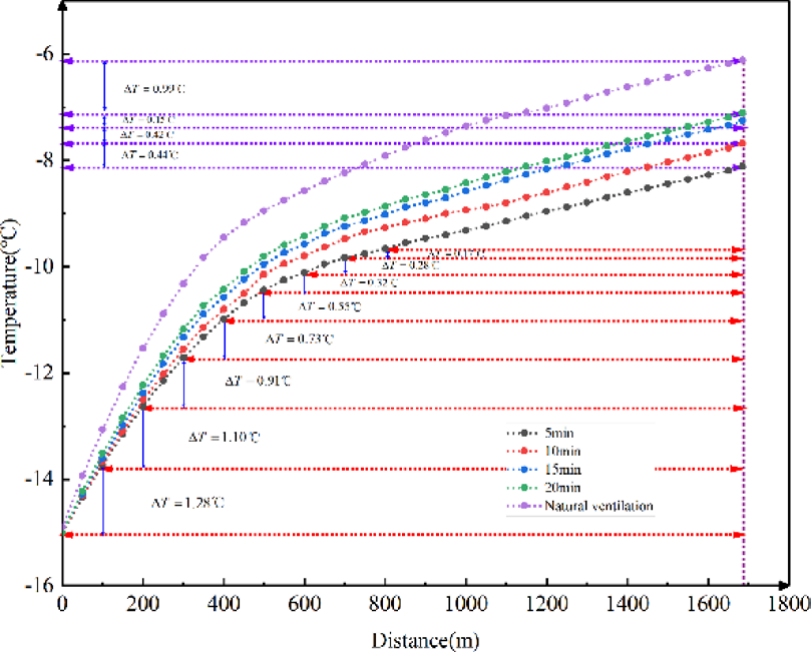
Longitudinal temperature field variations in the tunnel with different train frequencies.

As shown in Fig. 8, a comparison between the cases with and without trains passing through the tunnel reveals that the longitudinal temperature in the tunnel decreases significantly when a train is running. Therefore, the presence of a train in the tunnel has a substantial impact on the temperature field in the tunnel.

Overall, the temperature in the tunnel decreases as the train frequency increases, and the two are inversely proportional. When the interval between two trains exceeds 20 min, the reduction in the train frequency has little effect on the tunnel’s temperature field. However, when the interval is shorter than 20 min, the effect of increasing frequency on the temperature field inside the tunnel gradually becomes more pronounced.

Yan Gao et al.^11^ conducted a statistical analysis of the measured data from 11 cold-region tunnels in China and found that the temperatures at the arch top, inverted arch, arch waist, and side wall of the tunnels were all different. The temperature at the side wall of the same tunnel section was the lowest, while the temperature at the inverted arch was the highest. In the absence of insulation layers, the average temperature difference between the tunnel wall and the interface between the secondary lining and the primary lining was 2.24 ℃. When the temperature of the secondary lining wall inside the tunnel was lower than − 2.24 ℃, an insulation layer needed to be laid at this time.

To address the freezing problems in tunnels in cold regions, scholars from both domestic and foreign countries have conducted numerous studies^25–28^. For instance, Norway adopted the external lining technique. By leveraging the low thermal conductivity of air, this approach effectively inhibits the infiltration of cold air. In China, to tackle the ice formation issue in cold-region tunnels, a comprehensive array of technical measures has been employed. These include the utilization of insulating materials, the installation of anti-freezing doors, and the implementation of insulation drainage measures. Russia makes use of the heating pipeline technology. This technology raises the internal temperature of the tunnel, thus preventing ice formation in cold-region tunnels. The United States has established an insulation system. This system ensures that the tunnel drainage system remains unfrozen even under low-temperature conditions. France employs the isolation wall panel technology. By capitalizing on the low thermal conductivity of insulating materials, this technology prevents cold air from intruding into the tunnel.

### Impact of different surrounding rock temperatures

With the interval between two trains set to 15 min, external temperature to -15 ℃, and external wind speed to 2 m/s, the influence of train-induced airflow on the temperature field in the tunnel under the conditions of different surrounding rock temperatures was discussed.

**Fig. 9:**
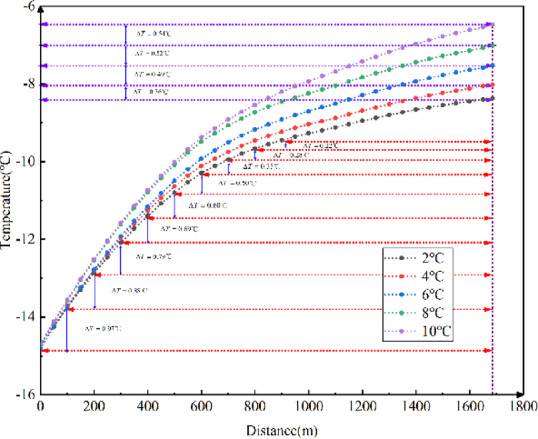
Longitudinal temperature field variations in the tunnel with different surrounding rock temperatures.

As shown in Fig. 9, the temperature field in the tunnel decreases as the surrounding rock temperature decreases, following a proportional relationship. For every 2 ℃ decrease in surrounding rock temperature, the temperature at the tunnel exits decreases by approximately 0.474 ℃. Therefore, the results indicate that lower surrounding rock temperatures lead to lower longitudinal temperatures in the tunnel.

**Fig. 10:**
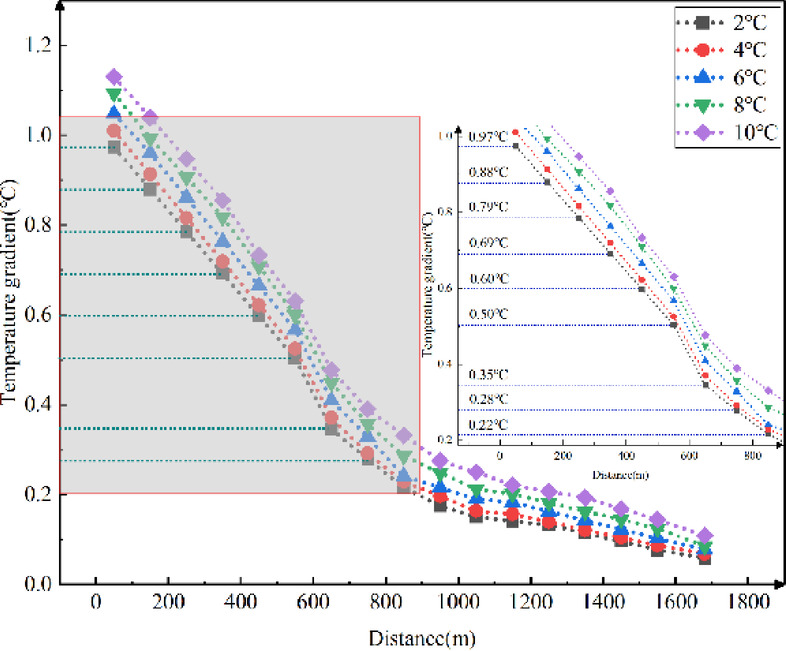
Longitudinal temperature gradients in the tunnel with different surrounding rock temperatures.

For every 100 m of longitudinal distance inside the tunnel, a point was selected to calculate the temperature difference between adjacent points, which was defined as the temperature gradient between the two points. As shown in Fig. 10, the temperature gradient decreases as the longitudinal distance rises. The lower the external temperature, the greater the temperature gradient at the tunnel entrance. The higher the surrounding rock temperature, the greater the temperature gradient at the tunnel entrance, and it decreases more rapidly with increasing longitudinal distance. The impact of surrounding rock temperature on the longitudinal temperature gradient in cold-region tunnels ranges from 0.059 ℃ to 1.13 ℃ for every 100 m.

### Impact of different external temperatures

With the interval between two trains set to 15 min, the ground temperature of surrounding rock to 6 ℃, and external wind speed to 2 m/s, how the external temperature affects the effect of train-induced airflow on the temperature field in the tunnel was discussed.

**Fig. 11:**
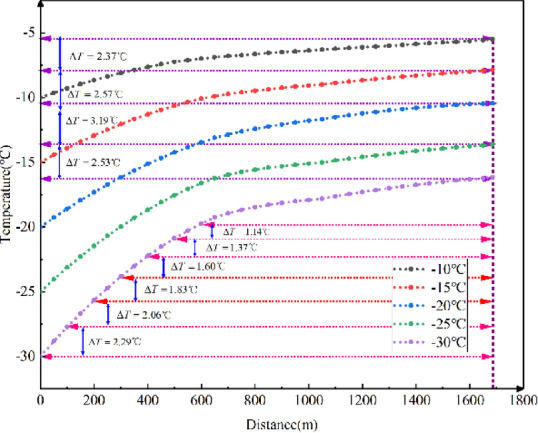
Longitudinal temperature field variations in the tunnel with different external temperatures.

As shown in Fig. 11, the temperature field in the tunnel decreases with the external temperature, following a proportional relationship. For every 2 ℃ decrease in external temperature, the temperature at the tunnel exits decreases by approximately 2.5 ℃; The lower the external temperature is, the greater the growth rate of the temperature field in the tunnel along the longitudinal distance. Therefore, the results indicate that the lower the external ground temperature, the lower the longitudinal temperature in the tunnel.

**Fig. 12:**
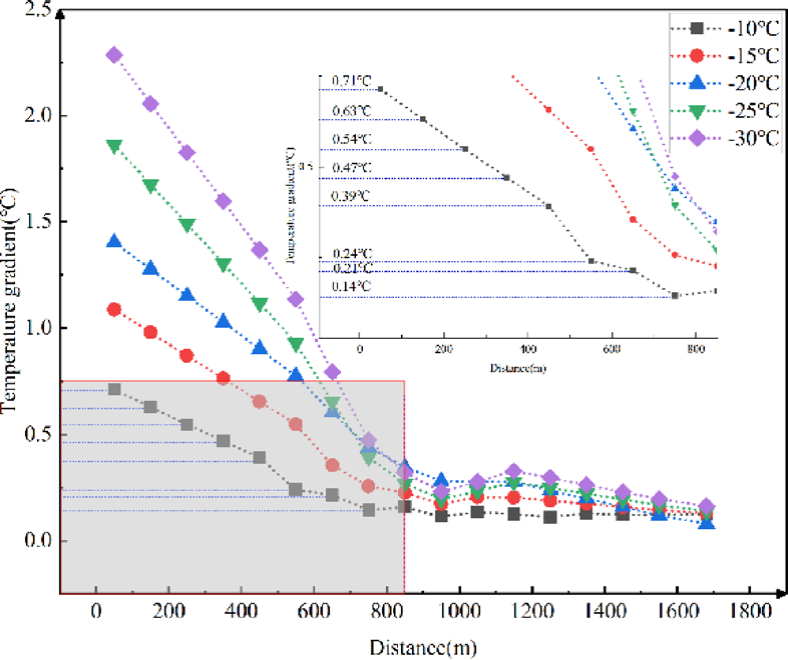
Longitudinal temperature gradients in the tunnel with different external temperatures.

Figure 12 illustrates that the temperature gradient decreases as the longitudinal distance increases. The lower the external temperature, the greater the temperature gradient at the tunnel entrance. This gradient decreases more rapidly with increasing longitudinal distance. The impact of external temperature on the longitudinal temperature gradient in cold-region tunnels ranges from 0.080 ℃ to 2.286 ℃ for every 100 m.

### Impact of different external wind speeds

With the interval between two trains set to 15 min, the ground temperature of surrounding rock to 6 ℃, and external temperature to -15 ℃, the impact of the variation of external wind speed on the temperature field in the tunnel was discussed.

**Fig. 13:**
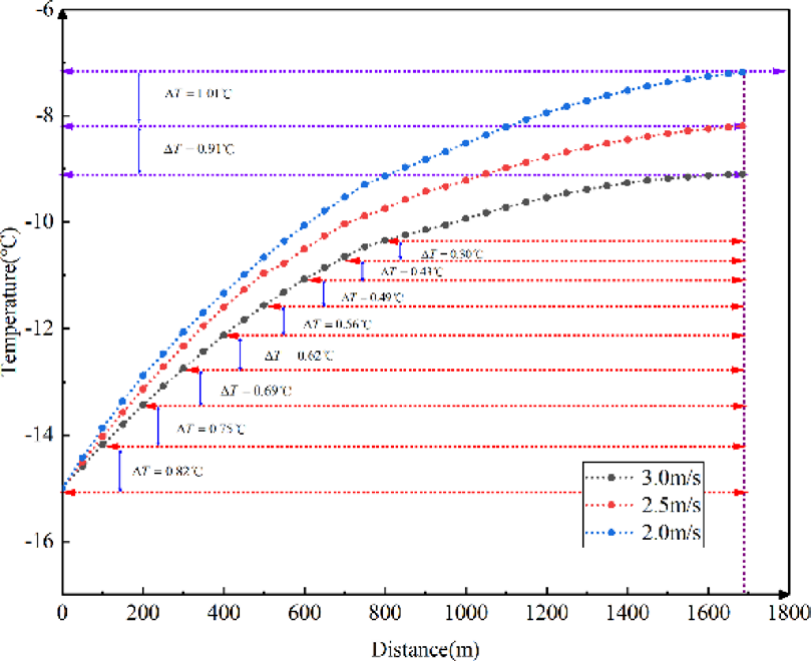
Longitudinal temperature field variations in the tunnel with different external wind temperatures.

As shown in Fig. 13, the temperature field in the tunnel decreases with increasing external wind speed, following an inverse relationship. For every 0.5 m/s increase in external wind speed, the temperature at the tunnel exit drops by approximately 2.5 ℃. Therefore, the results indicate that higher external wind speeds lead to lower longitudinal temperatures in the tunnel.

**Fig. 14:**
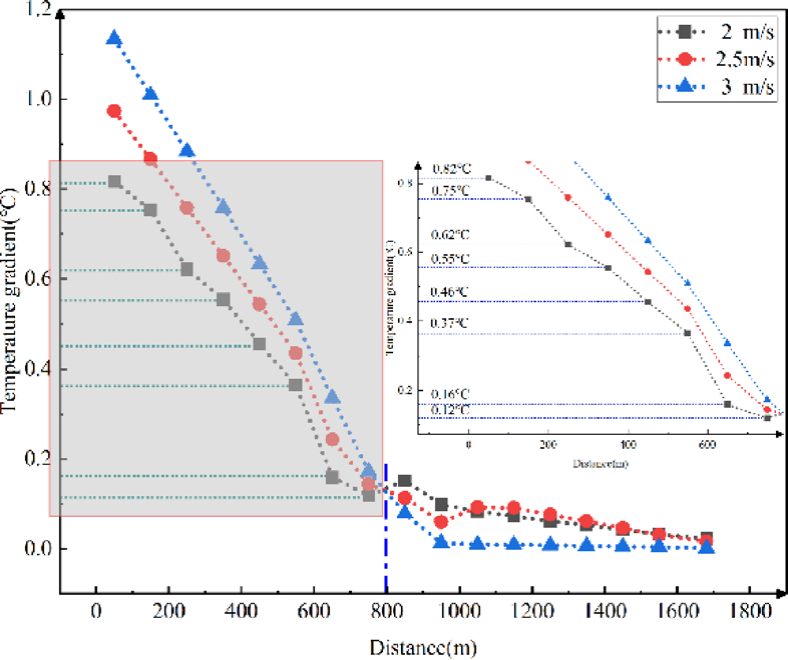
Longitudinal temperature gradients in the tunnel with different external wind speeds.

Figure 14 reveals that the temperature gradient decreases as the longitudinal distance increases. The higher the external wind speed, the greater the temperature gradient at the tunnel entrance. This gradient decreases more rapidly with increasing longitudinal distance. The impact of external wind speed on the longitudinal temperature gradient in cold-region tunnels ranges from 0.002 ℃ to 1.134 ℃ for every 100 m.

## Conclusion

(1) This study, based on unsteady flow theory, constructed the Navier-Stokes equations and proposed a theoretical calculation method for train-induced airflow that can represent the variation of the velocity of train-induced airflow in cold-region tunnels. The “equivalent wind speed” calculation method proposed has been validated through the comparison of theoretical, simulated, and experimental data, demonstrating its applicability in solving the impact of train-induced airflow on the temperature field in cold-region tunnels and providing a theoretical basis for related research.

(2) When the interval between two trains is less than 20 min, the average temperature in the tunnel decreases by approximately 1.71 ℃. The impact of train-induced airflow on the temperature field in cold-region tunnels is significant. The temperature in the tunnel decreases as the train frequency increases, showing an inverse relationship. Therefore, the impact of train-induced airflow on the tunnel temperature field should be fully considered in the insulation and anti-freeze design for cold-region tunnels.

(3) Higher surrounding rock temperature, lower external temperature, and greater external wind speed result in a higher temperature gradient at the tunnel entrance, with the gradient decreasing more rapidly as the longitudinal distance rises. The impact of the ground temperature of surrounding rock on the longitudinal temperature gradient in cold-region tunnels ranges from 0.059 ℃ to 1.13 ℃ for every 100 m, and the ranges for external temperature and external wind speed are 0.080 ℃ to 2.286 ℃ and 0.002 ℃ to 1.134 ℃, respectively.

## Supplementary Information

Below is the link to the electronic supplementary material.

Supplementary Material 1

## Data Availability

Data supporting the finding of this study are available upon requests. To obtain access to the data, please contact the corresponding author.
